# Combined Analyses of Bacterial, Fungal and Nematode Communities in Andosolic Agricultural Soils in Japan

**DOI:** 10.1264/jsme2.ME11281

**Published:** 2012-01-06

**Authors:** Zhihua Bao, Yoko Ikunaga, Yuko Matsushita, Sho Morimoto, Yuko Takada-Hoshino, Hiroaki Okada, Hirosuke Oba, Shuhei Takemoto, Shigeru Niwa, Kentaro Ohigashi, Chika Suzuki, Kazunari Nagaoka, Makoto Takenaka, Yasufumi Urashima, Hiroyuki Sekiguchi, Atsuhiko Kushida, Koki Toyota, Masanori Saito, Seiya Tsushima

**Affiliations:** 1 National Institute for Agro-Environmental Sciences, 3–1–3 Kannondai, Tsukuba 305–8604, Japan; 2 Soil Microbiology Research Team, National Agricultural Research Center, Ibaraki 305–8666, Japan; 3 National Agriculture and Food Research Organization National Agricultural Research Center for Tohoku Region, Arai, Fukushima City, Fukushima 960–2156, Japan; 4 National Agricultural Research Center for Hokkaido Region (NARCH), National Agriculture and Food Research Organization (NARO), 9–4 Shinsei-minami, Memuro-cho, Kasai-gun, Hokkaido 082–0081, Japan; 5 Graduate School of Bio-Applications and Systems Engineering, Tokyo University of Agriculture and Technology, Tokyo 184–8588, Japan; 6 Field Science Center, Graduate School of Agriculture Science, Tohoku University, 232–3 Yomogida, Naruko-onsen, Osaki, Miyagi 989–6711, Japan

**Keywords:** bacteria-fungi-nematode community, andosols, PCR-DGGE, two-way cluster analysis

## Abstract

We simultaneously examined the bacteria, fungi and nematode communities in Andosols from four agro-geographical sites in Japan using polymerase chain reaction-denaturing gradient gel electrophoresis (PCR-DGGE) and statistical analyses to test the effects of environmental factors including soil properties on these communities depending on geographical sites. Statistical analyses such as Principal component analysis (PCA) and Redundancy analysis (RDA) revealed that the compositions of the three soil biota communities were strongly affected by geographical sites, which were in turn strongly associated with soil characteristics such as total C (TC), total N (TN), C/N ratio and annual mean soil temperature (ST). In particular, the TC, TN and C/N ratio had stronger effects on bacterial and fungal communities than on the nematode community. Additionally, two-way cluster analysis using the combined DGGE profile also indicated that all soil samples were classified into four clusters corresponding to the four sites, showing high site specificity of soil samples, and all DNA bands were classified into four clusters, showing the coexistence of specific DGGE bands of bacteria, fungi and nematodes in Andosol fields. The results of this study suggest that geography relative to soil properties has a simultaneous impact on soil microbial and nematode community compositions. This is the first combined profile analysis of bacteria, fungi and nematodes at different sites with agricultural Andosols.

Soil is one of the most diverse habitats on earth and contains one of the most diverse assemblages of living organisms such as bacteria, fungi, nematodes and protozoa (9, 37). Soil biota play a vital role in the maintenance of soil fertility and productivity and is influenced by a number of factors, including soil properties (6, 15, 19, 21, 22, 28, 54) and anthropogenic activities (8, 23, 27, 43). Recently, spatial distance has recognized effects on microbial (4, 16, 20, 29, 46, 48) and nematode communities (12, 13, 47). For example, Green (20) described that spatial distance controls fungal community diversity on a large scale, and Fujimura *et al.*(16) reported that the site and soil properties affected the fungal community in forest soil. For bacterial communities, Ge *et al.*(18) reported that the sampling site had a greater effect on soil bacterial community diversity than other soil properties and bacterial diversity differed from the sampling scale. Robertson and Freckman (47) found a high density major feeding group of nematodes on a relatively small sampling scale in arable soil; however, little is known about the effect of soil properties at different sites on bacteria, fungi and nematode communities.

Although Andosol soil, derived from the geologic substrata of volcanic origin, is less than 1% of the world soil area, it is one of the most important soil groups for agricultural activities in Japan because it covers about 16.4% land surface and 46.5% arable upland fields (35). Several studies on microbial communities (51, 54, 57) and nematode composition (56) have been reported in arable soil, including the Andosol group in Japan. Most of these works, however, have been site-specific, limiting our understanding of the factors that influence soil microbial communities across regions.

One of the reasons for the paucity of soil biota studies on Andosol may be due to the limit of the available methodologies. Hoshino *et al.*(25, 26) established the DNA extract method for bacteria and fungi from Andosol using skim milk. Moreover, Morimoto and Hoshino (36) developed the standard PCR-DGGE (polymerase chain reaction-denaturing gradient gel electrophoresis) method in Japan, which enabled researchers in Japan to analyze soil samples in the same way. Concerning nematodes, Okada and Oba (44) developed the PCR-DGGE method. A well-established molecular biological approach, PCR-DGGE is now being used to gain a better understanding of the ecology of the soil microbial community (38, 39) and it has been used in various soil environments, including agricultural soils (40), grasslands (8, 21), plant rhizospheres (51), and paddy soil (1). In addition, the microbial community has been assessed by PCR-DGGE on a large scale (18).

An environmental DNA (eDNA) project with culture-independent molecular approaches was started in 2006 by the Ministry of Agriculture, Forestry and Fisheries in Japan to evaluate not only the physical and chemical properties of soils but also biological properties and to utilize their information for soil management. In this project, soil biodiversity analysis methods were developed with eDNA, which is directly extracted from soil. In addition, the ‘eDNA database for Agricultural soil (eDDSs)’ was constructed. In this project, we analyzed the relationship between biological properties and soil physicochemical properties or cultivation practice on a large scale using statistical analysis such as redundancy analysis and two-way cluster analysis.

The objectives of this study were to investigate the factors including soil properties and geographical sites affecting bacterial, fungal and nematode communities in Andosols in Japan.

## Materials and Methods

### Soil sampling

**Fig. 1:**
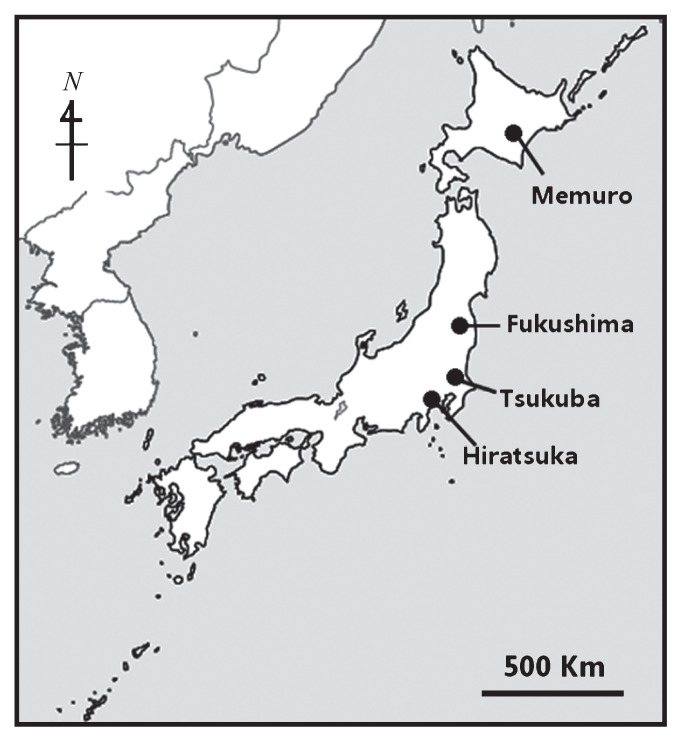
Sampling sites for soil bacterial, fungal and nematode communities in Japan.

In total, 32 bulk soil samples (8 samples per each site) were collected pre-cultivation from arable lands in Memuro (MM), Fukushima (FS), Hiratsuka (HT) and Tsukuba (TK), 4 locations distributed across eastern and northern Japan (Fig. 1 and Table 1). Eight samples were taken from eight independent plots in a field (56–240 m^2^) at each site. Each of the samples consisted of a mixture of at least 5 soil samples taken from the plow layer (0–15 cm in depth) of soil at different places within the plot pre-cultivation in 2007. The soils were classified as Andosols according to the FAO (Food and Agriculture Organization) classification system (14). All samples were sieved through a 2-mm sieve and divided into two parts: one was stored at 4°C for soil chemical analyses, and the other frozen at −80°C for molecular analyses. The soil characteristics are listed in Table 1. Total carbon (TC) and total nitrogen (TN) were determined by the Dry Combustion Method and available P (AP) was measured using the Truog method. Soil temperature (ST) was obtained from the “Soil Information Web viewer” (National Institute for Agro-Environmental Science, http://agrimesh.dc.affrc.go.jp/soil_db/). Spatial resolution of this map is 1 km (55)

### DNA extraction

For the bacterial and fungal PCR-DGGE, DNA was extracted from 0.4-g soil samples using a FastDNA SPIN Kit for Soil (Q-biogene/MP Biomedicals, Solon, OH, USA) following the manufacturer’s instructions with slight modification. Since it was difficult to extract DNA from Andosols, 60–160 μL of autoclaved 20% skim milk solution were added in the first step (36). For the nematodes, DNA was extracted from nematodes collected from soil samples according to the modified Baermann Funnel Method as previously described (44). Briefly, 300 individual nematodes from each soil sample were first concentrated on a polytetrafluoroethylene (PTFE) membrane (pore diameter 0.5 μm; Advantec, Tokyo, Japan) using a vacuum and then resuspended in 200 μL nuclei lysis solution (Promega, Madison, WI, USA) and transferred to a 2-mL homogenate microtube containing 0.1 g glass beads (0.1 mm in diameter) and four zirconium silica beads. Fifty microliters each of skim milk (20%) and EDTA (0.5 M, pH 8.0) were added and tubes were frozen at −80°C for at least 15 min. The frozen tubes were shaken on a homogenizer (FastPrep 100A; Thermo Electron, Waltham, MA, USA) for 155 s at a speed of 6.5 m/s to extract DNA. The DNA was then purified using the Wizard SV genomic DNA purification system (Promega) according to the manufacturer’s instructions and frozen at −80°C until further analysis.

### PCR-DGGE analysis

The DGGE analyses were performed using previously published procedures for bacteria, fungi (36) and nematodes (41). PCR of bacterial 16S rRNA genes was conducted using the universal primer set 968f-GC and 1378r (24). The PCR reaction mixture (50 μL) contained 5 μL of 10×PCR buffer, 0.2 μM of each primer, 1 U of KOD-plus (Toyobo, Osaka, Japan), 0.4 μM BSA (Takara Bio, Otsu, Japan), 1 mM MgSO_4_, 0.2 mM of each dNTP and 1 μL template DNA. The PCR program was as follows: initial denaturation at 94°C for 2 min, 34 cycles of denaturation at 94°C for 15 s, annealing at 55°C for 30 s and extension at 68°C for 30 s. The molecular marker for bacterial DGGE analysis (DGGE Marker III; Nippon Gene, Toyama, Japan) was used.

PCR of fungal 18S rRNA genes was conducted using primer sets NS1 and GCFung (33). The PCR reaction mixture (50 μL) contained 5 μL of 10×PCR buffer, 0.3 μM of each primer, 1 U KOD-plus, 0.4 μM BSA (Takara Bio), 1 mM MgSO4, 0.2 mM of each dNTP and 1 μL template DNA. The PCR program was as follows: initial denaturation at 94°C for 2 min, 30 cycles of denaturation at 94°C for 15 s, annealing at 55°C for 30 s and extension at 68°C for 30 s. The molecular marker for fungal DGGE analysis (DGGE Marker IV, Nippon Gene) was used.

PCR of nematode 18S rRNA genes was conducted using the primer sets SSU18A and SSU9R-GC (5). The PCR reaction mixture (25 μL) contained 5 μL of 5×PCR buffer, 0.5 μM of each primer, 0.6 units of Prime Star Polymerase HS (Takara Bio), 0.2 mM dNTPs and 10 μL nematode DNA solution. The PCR program was as follows: initial denaturation at 98°C for 3 min; 27 cycles of denaturation at 98°C for 10 s, annealing at 54°C for 15 s and extension at 72°C for 40 s. The molecular marker for nematode DGGE analysis (DGGE Marker V; Nippon Gene) was used.

The DGGE was performed using a DCode system (Bio-Rad Laboratories, Hercules, CA, USA). For the bacterial analysis, the conditions for separation were as follows: running condition set at 50 V at 58°C for 18 h in a 6% polyacrylamide gel with a denaturing gradient ranging from 50 to 70% (a gel with 7 M urea was designated as 100% gel according to Muyzer) (38). For fungal analysis, a 7% polyacrylamide gel with a denaturing gradient ranging from 20 to 45% was utilized, with the running condition set at 50 V at 60°C for 20 h. For nematode analysis, a 6% polyacrylamide gel with a denaturing gradient ranging from 20 to 50% was utilized, with the running condition set at 75 V at 60°C for 16 h. After electrophoresis, the gels were stained with SYBR Green I Nucleic Acid Gel Stain (Cambrex Bio Science, Rockland, ME, USA) for 30 min, and scanned with a Molecular Imager FX system (Bio-Rad Laboratories). The images were acquired with a Quantity One® image analysis system (Bio-Rad Laboratories) and stored as TIFF files. Band patterns were analyzed using GelCompar II software (version 4.0; Applied Maths, Kortrijk, Belgium) for Windows.

### Statistical analysis

The relative intensity from the gel band obtained by GelCompar was used in subsequent analyses, which eliminated the variation in band intensity caused by the difference in the amounts of PCR products loaded in the gel (52). For the nematode community, DGGE band data from the third to ninth positions from the top were used in our study, because the first and second marker bands may be associated with other eukaryotes, including ciliates (44). Principle components analysis (PCA) and redundancy analysis (RDA) for bacterial, fungal and nematode communities were performed using CANOCO (http://www.microcomputerpower.com/) for Windows, version 4.51. RDA was used to analyze the DGGE profile and environmental data such as soil characteristics, because detrended correspondence analysis (DCA) revealed that the data exhibited a linear, rather than a unimodal, response to the environmental variables (28). Monte Carlo permutation tests were based on 999 random permutations of the data. Normal curve tests were conducted using JMP version 5.0 (SAS Institute, Cary, NC, USA) for all variables before performing RDA, and AP values were log-transformed. To examine the relative effects of soil characteristics on bacterial, fungal and nematode community compositions, we analyzed each individual DGGE band of each soil organism that was significantly correlated (*P*<0.05) with environmental characteristics using a multiple regression method by JMP (version 5.0) and calculated the percentage of overall DGGE bands in each soil biota, respectively. Two-way cluster analysis was performed using PC-ORD version 5.03 (34) and the combined bacterial, fungal and nematode communities from each sample were revealed by DGGE profiles.

## Results

### Soil chemical characteristics

The properties of the soil samples collected at the four sites (MM, FS, HT, TK) are shown in Table 1. The available P (AP) concentration at the MM site was 50.3 mg P_2_O_5_ kg^−1^ and was significantly lower than at the FS (127.31 mg P_2_O_5_ kg^−1^), HT (191.50 mg P_2_O_5_ kg^−1^) and TK (350 mg P_2_O_5_ kg^−1^) sites. The soil pH values ranged from 6.1 to 5.7, and the TC (%) and TN (%) ranged from 4.7 to 1.6 and 0.34 to 0.18, respectively. The C/N ratios ranged from 9.1 to 20.5 and were significantly higher at MM (20.5) than at the FS (9.1), HT (10.0) and TK (12.0) sites.

### Bacterial, fungal and nematode communities

**Fig. 2:**
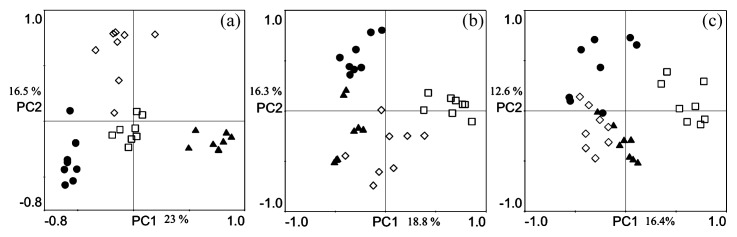
Principal component analysis (PCA) based on PCR-DGGE profiles of the bacterial (A), fungal (B) and nematode (C) communities. □, samples of MM site; ●, samples of FS site; ▲, samples of HT site; ⋄, samples of TK site.

The bacterial, fungal and nematode community structures obtained by the DGGE profiles were analyzed by PCA (Fig. 2). The PCA plots of the DGGE profiles explained 39.5%, 35.1% and 29% of the variance in bacteria, fungi and nematodes, respectively. There were trends toward clustering that were consistent with the geographic sites of the soils.

**Fig. 3:**
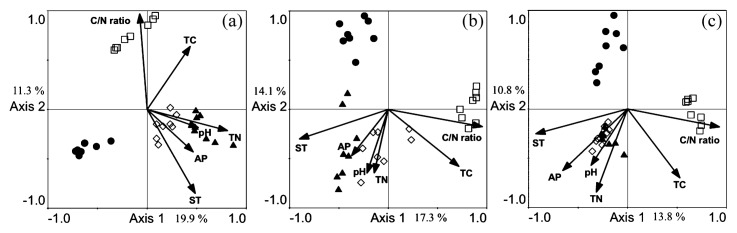
Redundancy analysis (RDA) ordination diagram of bacterial (A), fungal (B) and nematode (C) PCR-DGGE profiles, with environmental variables such as TC, TN, C/N ratio, pH, AP and ST. □, samples of MM site; ●, samples of FS site; ▲, samples of HT site; ⋄, samples of TK site.

RDA was also performed to reveal the relationships between bacterial, fungal and nematode communities and environmental variables (Fig. 3). RDA showed that site and environmental variables (Table 1) were the main factors differentiating communities. Together, axes 1 and 2 accounted for 31.2%, 31.4% and 24.6% of the total variation within bacterial, fungal and nematode communities, respectively. The three soil biota at the HT site were more strongly associated with TN and pH (Fig. 3). Similarly, the MM site had a positive correlation with the C/N ratio, but a negative correlation for AP and ST for all soil biota, and the FS site had a negative correlation with TC, TN and pH (Fig. 3).

The percentage of DGGE bands among all samples significantly correlated with environmental variables based on multiple regression for the three soil biota, shown in Table 2. A total of 59, 48 and 55 DGGE bands were detected in bacterial, fungal and nematode communities, respectively. For the bacterial community, TC, TN and C/N ratios were significantly correlated with 29% (17 bands), 31% (18 bands) and 39% (23 bands) of all bands, respectively. On the other hand, 17% (10 bands), 10% (6 bands) and 7% (4 bands) of all bands were correlated with ST, AP and pH, respectively. For the fungal community, ST was more correlated (33%) with the DGGE bands than the other environmental variables, and TC, TN and C/N ratios showed the same percent correlation (25%), while AP and pH showed correlations of 10% and 7%, respectively. For the nematode community, no environmental variables were related to more than 20% of DGGE bands. Both the C/N ratio and AP were related to 18% (10 bands) of DGGE bands, and ST, TC, TN and pH were related to 16% (9 bands), 16% (8 bands), 13% (7 bands) and 7% (4 bands) of DGGE bands. TC, TN and C/N ratios had greater effects on the microbial (bacterial and fungal) community composition than on the nematode community. ST and AP had greater effects on fungal and nematode communities than on other soil biota, respectively. Soil pH had a weak effect on all studied soil communities.

### Integrated combination of bacterial, fungal and nematode communities

**Fig. 4:**
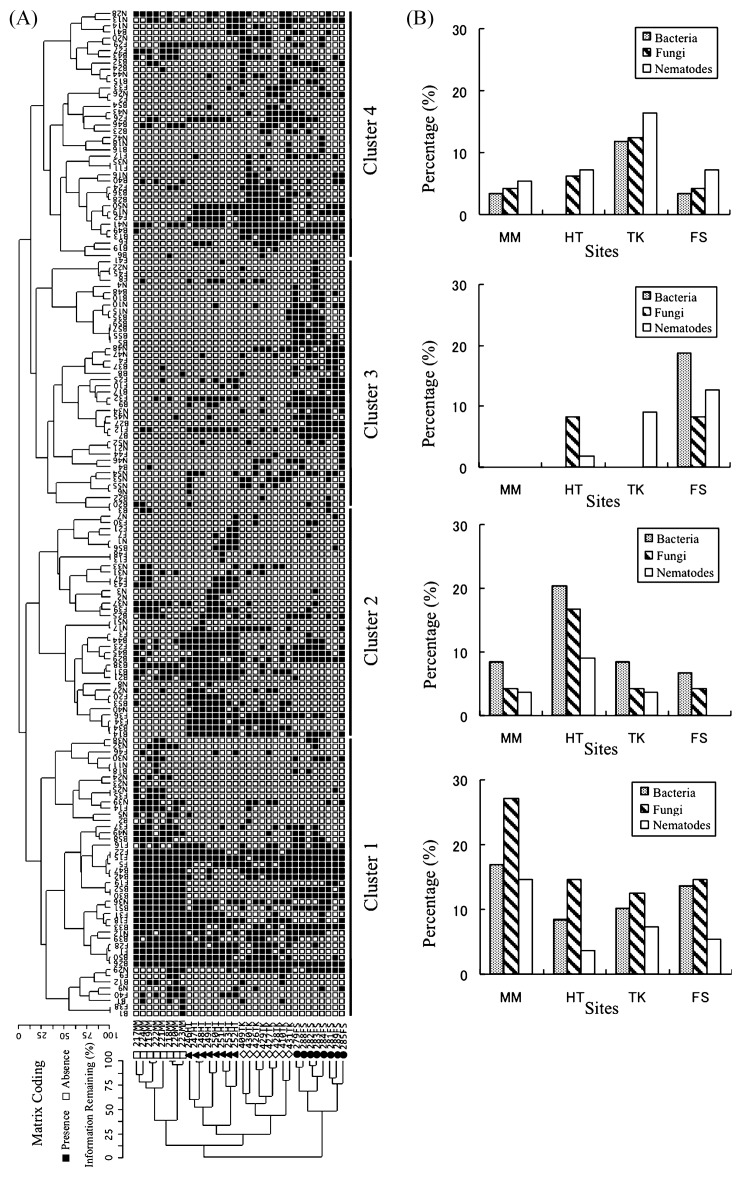
Two-way cluster analysis of combined DGGE profiles of bacterial, fungal and nematode communities. The results of cluster analysis of DGGE bands (left side) and PCR-DGGE profiles combining bacterial, fungal and nematode communities (lower side) are shown in (A). Black and white boxes in (A) indicate the presence and absence of DGGE bands in each sample, respectively. For each cluster obtained from cluster analysis of DGGE bands (left side shown in (A)), the percentages of the DGGE bands with high detection frequency, which were detected in more than 3 out of 8 soil samples, in each site (□, MM; ●, FS; ▲, HT; ⋄, TK) are shown in (B).

DGGE profile based on bacterial, fungal and nematode communities clearly showed that the 32 samples were classified into four sites by two-way cluster analysis (Fig. 4A). Site-specific clusters were constructed according to DGGE bands, including bacteria, fungi and nematodes (Fig. 4B). Cluster 1 represented frequent DGGE bands at the MM site; Cluster 2 contained DGGE bands that were restricted to the HT site; Cluster 3 represented DGGE bands that predominated at the FS site; Cluster 4 represented DGGE bands that predominated at the TK site. In all cases, Cluster 1 DGGE band appeared much more commonly at the MM site than DGGE bands in other clusters. Bacterial, fungal and nematode DGGE bands from the MM site were not detected in Cluster 3 and bacterial DGGE bands from the HT site were not detected in Clusters 3 and 4. Bacterial and fungal DGGE bands from the TK site did not appear in Cluster 3 and nematodes from the FS site were not detected in Cluster 2. These results indicate that there were 11 sets of detectable DGGE bands of bacteria, fungi and nematodes within the four main site-dependent clusters.

## Discussion

The relationship between the microbial community and environmental factors such as soil chemical properties and soil temperature was investigated at four agro-geographical sites with Andosols in Japan. This work is the first attempt to simultaneously investigate on a large scale the bacterial, fungal and nematode communities in arable soil, Andosol.

A key feature of our study was to collect soil samples from farmers’ fields with similar soil types (Andosols) at four different geographical sites. This was meant to minimize the effect of soil type on biological communities, as the effect of soil type on microbial communities has been previously reported (54).

Site characteristics and soil properties have recognized effects on microbial (16, 29) and nematode communities (12). If samples cluster by habitat, it can be concluded that soil communities are influenced by the contemporary environment (32). In this study, bacterial, fungal and nematode communities were strongly associated with geographical sites along with soil temperature and chemical gradients. In particular, different effects of soil characteristics and soil temperature were shown among communities of microbes (bacteria and fungi) and nematodes. Several studies on other soil types have also reported the effects of TC, TN and C/N ratios on microbial communities (2, 10, 16, 17, 31). In this study, TC and TN affected more fungal and bacterial communities than nematode communities, respectively (Table 2). This finding was consistent with the results of other small-scale studies that compared the effects of manure fertilizer, including rich TC and TN treatments on bacterial, fungal and nematode communities (11). That TC and TN affect bacterial and fungal communities more than nematode communities regardless of the study scale suggests that bacterial and fungal communities can be controlled more easily than nematode communities. The effect of soil warming on bacterial and fungal communities has also been previously reported (16, 53, 62), but the impacts on soil microbial communities varied and were often unpredictable. In this study, ST strongly affected all three soil biotas, although the effects of ST were higher on fungal communities than on bacterial and nematode communities. This suggests that the effect of meteorological conditions on soil bacterial, fungal and nematode communities cannot be neglected in large-scale studies.

Phosphorus application can lead to increased soil soluble P and impact fungal and bacterial activities (3). Andosols generally have low levels of AP caused by strong P adsorption capacity (58). The higher levels of AP (50.3–350 mg P_2_O_5_ kg^−1^) among our sites may have been caused by the application of fertilizer (60). The effect of AP on the microbial community has also been previously reported (16, 31). For example, Lauber *et al.*(31) suggested that extractable soil P may be an important regulator of the large-scale biogeographical patterns exhibited by fungal communities in forest soils. Among the environmental variables such as TC, C/N ratio and ST, AP had the greatest effect on nematodes and the least effect on bacterial and fungal community compositions, even though there was a large gradient of AP among the sites in this study. This suggests that, compared to other factors, soil microbial communities are not sensitive to the amount of AP, at least in the range of 50.3 to 350 mg P_2_O_5_ kg^−1^.

On the other hand, several studies have reported that soil pH is the most important factor affecting bacterial communities on a large scale (10, 15, 22, 49, 57). In these studies, different soil types were sampled widely and there were large differences in the soil pH among the samples. For instance, Terahara *et al.*(57) reported that soil pH (4.2–7.6) had a greater effect on the bacterial community than other environmental variables on a wide scale in several soil types including Andosols in Japan. In contrast, in our study, there were no significant differences in soil pH (5.7–6.1) among the sampling sites, and soil pH had lower effects (<7%) on the community compositions of the examined soil biota than other environmental factors. This may be due to the narrow pH range among their samples (5.7–6.1) compared with others (*e.g.*, 4.2–7.6 by Terahara (57)). These results suggest that pH had less effect on the composition of the microbial community, at least in the range of 5.65 to 6.10 in Andosol.

Combined analyses of the bacterial, fungal and nematode communities suggested the existence of site-specific groups of bacteria, fungi and nematodes (Fig. 3). This finding is also supported by the results of RDA regarding each microbial community (Fig. 3). Some of the combined communities seem to be dependent on sites that may present favourable conditions for coexistence among specific species of bacteria, fungi and nematodes. Several studies have reported that the distribution of microbial taxa, such as *Pseudomonas*, *Rhodopseudomonas* and *Bradyrhizobium* species, was related to geographical distance (7, 16, 42, 50). Despite similar habitat types, different nematode taxa may occur depending on the geographical region, as previously reported (13). The results of this study, and comparison with others, suggest that geography has a simultaneous impact on soil microbial and nematode community compositions.

Within the soil, the consumption of microbes by other soil fauna is likely to be an important driving factor of soil microbial community structure (59). For instance, bacterial-and fungal-feeding nematodes are generally involved in the regulation of bacteria and fungi, respectively (11, 30, 61), and promote the development of plant disease caused by fungi (45). Although nematodes were not classified based on microbial feeding habits in this study, this suggests that site-specific bacteria and fungi are likely related to their site-specific nematode-feeding group. The bacterial, fungal and nematode DGGE band groups obtained from two-way cluster analysis (Fig. 4A and B) may support the above studies. Further study is needed to address in detail whether the results shown in Fig. 4 are due to an interaction between microbial and microbial-feeding nematodes or the effect of geographical sites and environmental variables on soil biota alone. For example, it will be necessary to monitor community dynamics and interactionematodes.

In conclusion, the present study revealed that the compositions of soil bacterial, fungal and nematode community simultaneously had an effect according to geographical sites relative with soil properties and site-specific DGGE bands of microbial and nematode corresponding to the four sites. Further research is needed to clarify whether agro-geographical sites with various soil types have specific DGGE bands. The eDNA database will facilitate the more systematic integration of microflora and macrofauna into soil ecological studies and will allow for analysis of large numbers of soil samples to address multitrophic interaction. In addition, identification of site (region)-specific indicator organisms will become more likely as the number of DGGE bands or high-frequency DGGE bands at sites or regions increases through the collection of a large number of soil samples. This study is a first step in expanding our knowledge of key topics in agricultural soil ecosystems and communities.

## Figures and Tables

**Table 1:** Environmental data obtained from four Andosol sampling sites

Sample name	Sampling location (Prefecture)	Latitude/longitude	Sampling dates	TC (%)*	TN (%)*	C/N ratio	pH (H_2_O)	AP* (mg P_2_O_5_ kg^−1^)	ST* (°C)
MM	Memuro (Hokkaido)	N42°53′21″/E143°4′32″	2007.7.18	4.7 (±0.3)**	0.25 (±0.08)	20.5 (±0.5)	5.8 (±0.1)	50.3 (±10.7)	8.5
FS	Fukushima (Fukushima)	N37°42′37″/E140°23′29″	2007.8.28	1.6 (±0.3)	0.18 (±0.02)	9.1 (±0.5)	5.7 (±0.2)	127.3 (±131.1)	13.8
HT	Hiratsuka (Kanagawa)	N35°21′04″/E139°16′53″	2007.9.2	3.4 (±0.3)	0.34 (±0.05)	10.0 (±1.0)	5.9 (±0.2)	191.5 (±37.6)	16.7
TK	Tsukuba (Ibaraki)	N36°01′40″/E140°06′01″	2007.5.14	4.1 (±0.8)	0.34 (±0.05)	12.0 (±1.0)	6.1 (±0.3)	350.0 (±160.5)	15.5

* TC, total carbon; TN, total nitrogen; AP, available P; ST, soil temperature.

** Standard error of the mean (SEM) is shown in parentheses.

**Table 2:** Percentage of DGGE bands significantly correlated with environmental variables in overall DGGE bands of bacterial, fungal and nematode communities by multiple regression (*P*<0.05)

	Number of DGGE bands appeared in all samples	Percentage (%) of DGGE bands correlated to environmental variables (No. of DGGE bands)					

		TC	TN	pH	C/N ratio	ST	AP
Bacteria	59	29 (17)	31 (18)	7 (4)	39 (23)	17 (10)	10 (6)
Fungi	48	25 (12)	25 (12)	6 (3)	25 (12)	33 (16)	15 (7)
Nematode	55	15 (8)	13 (7)	7 (4)	18 (10)	16 (9)	18 (10)
